# Bacterial Adhesion on Soft Surfaces: The Dual Role of Substrate Stiffness and Bacterial Growth Stage

**DOI:** 10.3390/microorganisms13030637

**Published:** 2025-03-11

**Authors:** René Riedel, Garima Rani, Anupam Sengupta

**Affiliations:** 1 Physics of Living Matter Group, Department of Physics and Materials Science, University of Luxembourg, 162 A, Avenue de la Faïencerie, 1511 Luxembourg, Luxembourg; 2 Institute for Advanced Studies, University of Luxembourg, 2, Avenue de l’Université, 4365 Esch-sur-Alzette, Luxembourg

**Keywords:** bacteria, biofilms, surface stiffness, adhesion, force–displacement spectroscopy, AFM, phenotypic diversity

## Abstract

The surface adhesion and stiffness of underlying substrates mediate the geometry, mechanics, and self-organization of expanding bacterial colonies. Recent studies have qualitatively indicted that stiffness may impact bacterial attachment and accumulation, yet the variation in the cell-to-surface adhesion with substrate stiffness remains to be quantified. Here, by developing a cell-level force–distance spectroscopy (FDS) technique based on atomic force microscopy (AFM), we simultaneously quantify the cell–surface adhesion and stiffness of the underlying substrates to reveal the stiffness-dependent adhesion of the phototrophic bacterium *Chromatium okenii*. As the stiffness of the soft substrate, modeled using a low-melting-point (LMP) agarose pad, was varied between 20 kPa and 120 kPa by changing the agarose concentrations, we observed a progressive increase in the mean adhesion force by over an order of magnitude, from 0.21±0.10 nN to 2.42±1.16 nN. In contrast, passive polystyrene (PS) microparticles of comparable dimensions showed no perceptible change in their surface adhesion, confirming that the stiffness-dependent adhesive interaction of *C. okenii* is of a biological origin. Furthermore, for *Escherichia coli*, the cell–surface adhesion varied between 0.29±0.17 nN and 0.39±0.20 nN, showing a weak dependence on the substrate stiffness, thus suggesting that stiffness-modulated adhesion is a species-specific trait. Finally, by quantifying the adhesion of the *C. okenii* population across different timescales, we reported the emergent co-existence of weak and strongly adherent sub-populations, demonstrating diversification of the adherent phenotypes over the growth stages. Taken together, these findings suggest that bacteria, depending on the species and their physiological stage, may actively modulate cell-to-surface adhesion in response to the stiffness of soft surfaces. While the surface properties, for instance, hydrophobicity (or hydrophilicity), play a key role in mediating bacterial attachment, this work introduces substrate stiffness as a biophysical parameter that could reinforce or suppress effective surface interactions. Our results suggest how bacteria could leverage stiffness-dependent adhesion and the diversity therein as functional traits to modulate their initial attachment to, colonization of, and proliferation on soft substrates during the early stages of biofilm development.

## 1. Introduction

From living tissues to biomedical scaffolds, bacteria attach to and colonize a wide range of surfaces, spanning orders of magnitude of stiffness [1,2]. Once attached, the subsequent growth and accumulation of surface-associated bacterial populations are underpinned by mechanotransduction, ultimately leading to well-developed biofilm structures [3,4,5,6]. Surface sensing and the mechanoresponse during the initial stages of biofilm development [7,8,9,10] are crucial to the long-term fate of biofilms. At the scale of individual cells, the surface energy of the underlying substrates, i.e., their hydrophobicity and hydrophilicity, influences bacterial adhesion [11,12,13,14,15]. In addition, van der Waals and electrostatic forces can regulate cell–surface interactions [16,17], thereby influencing biofilm growth. Recent results have also taken into account the role of local osmotic pressure and poroelastic characteristics of substrates in controlling biofilm growth and morphology [18], as well as drying of biologically relevant sessile droplets [19,20].

In recent years, multiple studies have indicated the possible role of substrate stiffness in bacteria–surface adhesion. Most of these studies have been conducted on agarose and poly-ethylene glycol (PEG) hydrogels [5,14,21] and on polymeric substrates, including poly-dimethylsiloxane (PDMS) [22,23,24] and composite thin films [25,26]. Although different bacterial species have been covered in these studies, a generalized framework—capturing the impact of stiffness on bacterial adhesion—is yet to emerge, in part due to observable inconsistencies in the reported trends [22,27,28]. Traditionally, agarose, a linear polymer composed of alternating D-galactose and 3,6-anhydro-L-galactopyranose monomers [29], has frequently been used in the study of bacterial growth and biofilm assays. Agarose has a high gel strength, transparency, [30], and non-toxicity, making it suitable for investigating bacterial adhesion, growth, and self-organization on such surfaces [5,21,31,32]. More recently, low-melting-point (LMP) agarose has gained attention as a special form of agarose [33,34,35], where the gelling temperature is decreased due to chemical modifications, for example, via hydroxyethylation [36]. The ability to form thermoreversible gels at low temperatures makes LMP agarose the preferred choice when gentle gelation conditions are desired, for instance, for bacterial assays [6,37,38], the encapsulation of heat-sensitive biomolecules [39], tissue engineering scaffolds [39], drug delivery systems [40,41], and 3D cell culture models [42].

Despite the growing relevance of LMP agarose in bacterial studies, thorough quantification of its mechanical properties is largely lacking. The bulk and local elasticity, stiffness, and adhesion of the substrates are of particular interest in microbial ecology, as they not only influence the physiology and behavior of the cells but also impact the biophysical underpinnings of bacterial growth, feedback, and emergent traits [43,44,45]. Elasticity is a fundamental mechanical property that characterizes the ability of a material to deform under an applied force and return to its original shape upon the removal of the force. Various techniques, including bulk rheology and local atomic force microscopy (AFM), have been employed to investigate the elastic properties of such soft gels to understand their behavior under different loading conditions [46,47,48]. Adhesion, the ability of a material to stick to another material, may emerge due to mechanical interlocking or various physico-chemical interactions between surface molecules [49]. In the context of bacterial adhesion, diverse species- and trait-specific attachment mechanisms have been proposed, which ultimately underpin the growth and dynamic self-organization into bacterial colonies [5,32,50,51,52]. While recent studies have indicated that bacterial attachment and accumulation could be impacted by the stiffness of the underlying substrates, currently, we lack methodologies that could allow for simultaneous measurements of both parameters. Furthermore, how stiffness-dependent adhesion evolves with the age of a bacterial population remains unexplored. As described in the aforementioned studies, the impact of stiffness on cell–surface adhesion has only received qualitative treatment so far, either via estimation of the surface density of the adhering cells or via retention assays, which estimate the relative fraction of leftover cells following a washing protocol. Quantification of the cell–surface adhesion forces, alongside concomitant measurement of the underlying substrate stiffness, is currently lacking.

Motivated by the gaps in our current understanding, here, we investigate the mechanical properties of LMP agarose gels prepared using different concentrations of LMP agarose in Lysogeny broth (LB) medium, a nutrient-rich liquid used to grow bacteria. We measure their elasticity according to their Young’s moduli and adhesion, both native and with respect to different bacterial species, by means of a cellular force–distance spectroscopy (FDS) technique based on atomic force microscopy (AFM) using LB medium and deionized (DI) water. Focusing on the Gram-negative bacterial species *Chromatium okenii*, we measure its cell–surface adhesion to LMP agarose and analyze how adhesion develops over its physiological growth stages. We compare our results with those for another Gram-negative species, *Escherichia coli*, and we assess the possibility of active modulation of the cell adhesion by contrasting the bacterial experiments with the adhesion of passive polystyrene beads with 5 μm and 20 μm diameters to LMP agarose substrates. Overall, our findings reveal that bacteria, depending on the species and their physiological state, can actively modulate cell-to-surface adhesion as a response to changes in the stiffness of the underlying soft surfaces. In doing so, bacteria may harness adherent phenotypes within a population to initiate attachment and optimize their proliferation strategies on soft substrates across a range of stiffnesses.

## 2. Experiments

### 2.1. Force–Distance Spectroscopy of Bacterial Cells on Soft Surfaces

By means of a Nanoscope^®^ V controller (Veeco, Plainview, NY, USA) atomic force microscope with a Multimode 8 scanner (Bruker, Billerica, MA, USA) [53], force distance curves were measured on the pure agarose gels using probes with a spherical SiO_2_ tip (3.5 μm diameter, 0.2 N/m force constant, CP-CONT-SiO, nanoandmore GmbH, Wetzlar, Germany). To measure cell adhesion, tipless cantilevers (0.2 N/m force constant, TL-CONT, nanoandmore GmbH, Germany) were used. The measurements were performed within fluid (deionized (DI) water or LB medium) using a fluid chamber with inlets and rubber sealing. The deflection sensitivity was determined beforehand from the slope of the retract curve in a force–distance spectroscopy measurement performed in the measurement fluid on a silicon surface. To translate the deflection into forces, the force constant measured using the nanoandmore GmbH was used. After removing the coverslip, the prepared sample holder was placed in the atomic force microscope, and a droplet of the measurement fluid was transferred onto the surface of the agarose gel. The prepared fluid chamber was installed and filled with measurement fluid through the inlets using syringes. Subsequently, the cantilever was manually brought close to the surface but was not brought into contact with it. During this procedure, the horizontal position of the cantilever was moved to the center position of the region of interest. After visually approximating close proximity between the tip and the surface, the probe was false engaged (i.e., tip–surface contact was simulated), and subsequently, the tip was manually edged towards the sample by means of step motor control and the force–distance curves continuously measured until an increase in the slope was detectable [54]. For the cell and microparticle adhesion measurements, the tip of the cantilever was first positioned close to regions of cell accumulation. A cell/microparticle was picked up by the tipless cantilever and used as the cantilever tip. Subsequent measurements were executed in a cell/microparticle-free region on the sample surface. We confirmed the presence and viability of bacterial cells on the tip of the cantilever using a combination of bright-field imaging and subsequent growth assays on an agarose pad. An in-built bright-field optical microscope on the AFM stage allowed us to visualize the bacterial cells on the cantilevers. The viability of the cells attached to the cantilevers was confirmed by bringing them in contact with nutrient-rich agarose pads. Upon contact, the cells were transferred to the agarose pad, on which they then grew (shown in the insets, with a scale bar of 10 μm). This confirmed that the bacteria present on a cantilever was viable. Force spectroscopy curves were recorded at 16 × 16 equidistant spots around the initial point of tip–surface contact. The distance between adjacent spots was 500 nm. The measurements were repeated at least 4 times on the same sample. The maximum applied force did not exceed 10 nN.

### 2.2. Optical Microscopy

To ensure that cells and microparticles were present on the tipless cantilever, the sample holder, as well as the tip holder, were investigated under an optical microscope (Eclipse LV100N POL, Nikon Corporation, Tokyo, Japan) in reflected-light mode right after the measurement and careful removal of excess liquid using a soft absorbent tissue.

### 2.3. Evalution of the Force–Distance Spectroscopy Data

**Figure 1:**
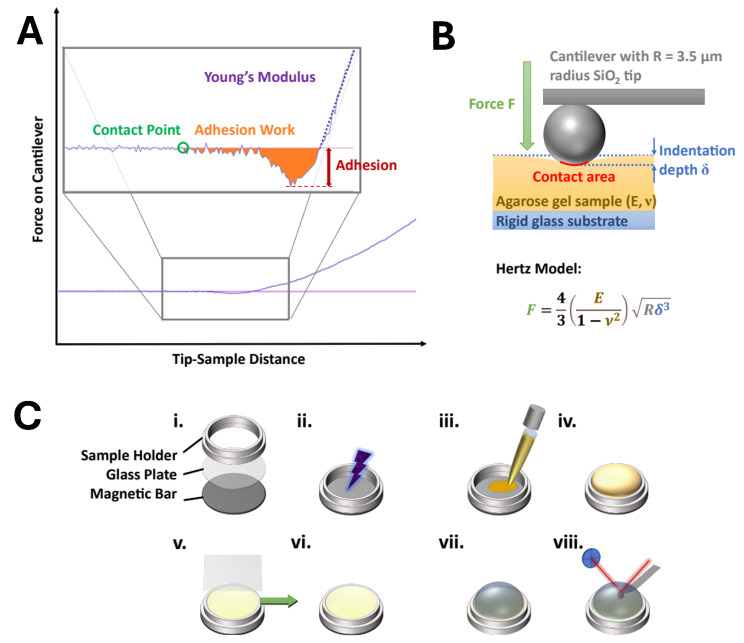
Experimental evaluation and setup. (**A**) Example force–distance curve. The inlet shows an enlargement of the dashed area. The adhesion force is extracted from the lowest force relative to the baseline. Young’s modulus is calculated from the slope of the curve. The maximum indentation force was 100 nN. (**B**) The Hertz model when a solid sphere with a radius *R* indents into a sample with Young’s modulus *E* and Poisson’s ratio *v*. (**C**) Sample preparation: The sample holder consists of a magnetic bar to immobilize the holder on the AFM scanner, a glass plate at the bottom, and the sample holder’s confinement (i). The glass is plasma-treated (ii) for 45 s. Agarose gel is heated up to liquefaction and then transferred onto the sample holder (iii) until it is completely filled (iv). While the gel is still liquid, a glass coverslip is placed onto the sample holder while avoiding the entrapment of air (v). The sample is allowed to gelify and cool down for 30 min. Before measurement, the coverslip is removed horizontally (v, vi). The sample is placed onto the atomic force microscope, and the measurement liquid is injected (vii). After 15 min of relaxation time, the measurement begins (viii).

AtomicJ (v 2.3.1, Pawel Hermanowicz) was used to extract the measured values from the recorded data files and process them by applying different theoretical models of the contact mechanics. A typical force–distance curve is shown schematically in Figure 1A. It consists of a region of no contact between the tip and the substrate and a region of indentation. These two regions are connected by the contact point $z_{c}$. For proper calculation, precise determination of the position of the contact point is indispensable. Contact mechanics models are applied to the contact area in the force–distance curves. They are based on the Hertz model [55], which describes the interaction between two spheres or a sphere and an endless plane surface (see Figure 1B). According to Hertz, the generalized force–indentation relation can be described as follows:

(1) $$F=\lambda \delta^{\beta }$$

where *F* is the applied force, δ is the indentation depth, and λ and β are values that depend on the underlying model. For the basic Hertz model of the indentation of a spherical body into a plane surface, β is 3/2, and λ is given by the following equation:

(2) $$F=\frac{4ER^{1/2}}{3(1-\upsilon^{2})}\delta^{3/2}$$

Here, *E* is the modulus of elasticity, *R* is the radius of the tip, and υ is the Poisson ratio. The latter is assumed to be ≈0.5 for very soft samples.

The Hertz model is a very basic model that does not consider the adhesion forces and their subsequent enhancement of the contact area. Derjaguin, Muller, and Toporov (DMT) [56], as well as Johnson, Kendall, and Roberts, JKR [57], have created extended models in parallel based on the Hertz model to incorporate the adhesion forces. These two models represent the limit values for the true value of elasticity. While the DMT model is more accurate for harder samples with low adhesion, soft samples with higher adhesion are better described using the JKR model.

In this paper, we therefore focus on evaluation using the JKR model via the following three equations:

(3) $$\delta =\frac{\alpha_{\mathrm{JKR}}^{2}}{R}-\frac{4}{3}\sqrt{\frac{\alpha_{\mathrm{JKR}}F_{\mathrm{ad}}}{RK}}$$

(4) $$\alpha_{\mathrm{JKR}}={\left[\frac{R}{K}{\left(\sqrt{F_{\mathrm{ad}}}+\sqrt{F+F_{\mathrm{ad}}}\right)}^{2}\right]}^{\frac{1}{3}}$$

(5) $$F_{\mathrm{ad}}=\frac{3}{2}\pi \gamma R$$

(6) $$K=\frac{4E}{3(1-\upsilon^{2})}$$

where $\alpha_{\mathrm{JKR}}$ is the JKR-extended contact area between the tip and the sample, $F_{\mathrm{ad}}$ is the tip–sample adhesion force, *K* is the elastic constant of the sample, and γ is the interfacial energy. The adhesion force, $F_{ad}$, is evaluated from the minimum point of the force–distance curve relative to its baseline.

*E* can hence be calculated by plotting δ versus *F* and adjusting *K* to minimize the least square error between the resultant plot and the measured data, starting from the point of tip–sample contact. The latter is ascertained numerically for the best fit. More comprehensive details on the evolution of force spectroscopy data have been well described by the works of Lin, Dimitriadis, and Horkay [58]. The evaluated data are represented as boxplots without whiskers. The inner 50% of the measured values sorted in ascending order is represented by a colored area containing the median as a horizontal line. Outliers were identified as data points that lay below $Q_{1}-1.5*IQR$ or above $Q_{3}+1.5*IQR$, where $Q_{1}$ and $Q_{3}$ are the first and third quartiles, respectively, and IQR is the interquartile range ($Q_{3}-Q_{1}$). Outliers are not shown here. In addition, the average value is shown as a point and the standard deviation as an error bar.

### 2.4. Low-Melting-Point Agarose Gel Preparation

Agarose gels of different concentrations (see Table 1) were prepared by slowly dissolving the respective quantity of low-melting-point agarose gel (agarose, LMP, preparative-grade for large fragments (>1000 bp), Promega, Madison, WI, USA)) into 5 mL of LB medium in an autoclaved glass tube with occasional stirring on a heat plate (50 °C). When the powder was completely dissolved, the solution was left to cool down to room temperature and then stored in a fridge (at 4 °C). Further details on the preparation of the agarose samples can be found in Ref. [52].

The steps for preparing the experimental samples are shown in Figure 1C. A ring-shaped sample holder with an inner diameter of 10 mm and a height of 2 mm was glued onto a glass coverslip with a diameter of 12 mm so that no glue was inside the ring. A magnetic plate was glued to the other side of the coverslip. The whole setup was dried for 24 h and used as the AFM sample holder. Prior to the sample preparation, the agarose gel was taken from the fridge and slowly heated on a heater plate. In order to increase the hydrophilicity of the sample holder glass, its surface was plasma-treated for 45 s using a plasma torch (Mode BD-20, Electro-Technic Products, Inc., Chicago, IL, USA). Then, 200 μL of the warm and viscous agarose gel was transferred into the sample holder. A coverslip was placed onto the sample holder to enclose the agarose gel, avoiding any air being trapped inside. The agarose gel was left to cool down for 30 min, and then the coverslip was slowly and horizontally removed just before the measurement. The topographies of two samples with 2.2% agarose were measured (in an LB medium environment and in a water environment) using a Nanoscope^®^ V controller (Veeco) atomic force microscope with a Multimode 8 scanner in Tapping mode using probes with a spherical SiO_2_ tip (3.5 μm diameter, 0.2 N/m force constant, CP-CONT-SiO, nanoandmore GmbH, Germany).

### 2.5. The Adhesion of Polystyrene Beads onto the LMP Agarose Gel

The polystyrene microparticles with 5 μm and 20 μm diameters were purchased from microparticles GmbH, Germany (product numbers PS/Q-R-KM650 and PS/Q-R-KM636 respectively). The stock solutions were diluted by a factor of $1\times 10^{-3}$ until single particles were visible on the substrate after sedimentation. The agarose gel sample was prepared as described above. After the removal of the coverslip from the agarose gel sample, a drop of the microparticle solution was placed onto the agarose gel sample. The microparticles were allowed to settle for 30 min before the drop was carefully removed. Subsequently, the force–distance curves were recorded as described above. This procedure was repeated three times. In order to measure the adhesion of the passive particles, the same measurements were repeated with agarose gel based on DI water. For each agarose concentration and particle size, the procedure was repeated three times.

### 2.6. Preparation of the Bacterial Assays

The forces of the adhesion of two different cell species, *Escherichia coli* (strain NCM 3722 ΔmotA, referred to as *E. coli*) and *Chromatium okenii* (referred to as *C. okenii*), to LMP agarose gel of different concentrations (1.5%, 2.2%, and 3.1%) were determined.

*E. coli* was stored in a freezer at −80 °C. Agar plates were prepared by dissolving 16 g of LB-agar (LB-Agar (Luria/Miller), CARL ROTH, Karlsruhe, Germany) into 400 mL of deionized water and subsequent autoclaving the mixture and storing it at 4 °C. The *E. coli* cultures were prepared using the in-house protocols discussed in Refs. [31,52]. The cells were taken out of the freezer and streaked onto an agar plate in a sterile environment at room temperature. The agar plate was placed in an incubator for 48 h at 30 °C (Infors HT Ecotron, Bottmingen, Switzerland). Subsequently, a single cell island was transferred from the agar plate into 8 mL of LB medium using a bacterial loop in a sterile environment. The cell solution was placed in the incubator at 30 °C and shaken at 180 RPM over 24 h. Thereafter, 200 μL of this cell solution was transferred into a 2 mL culture tube (Eppendorf, Hamburg, Germany) containing the LB medium and kept in an incubator at 30 °C under continuous shaking at 180 RPM. After 160 min, the cell suspension was centrifuged at 2000 rpm for 5 min. With the exception of 100 μL retained, the centrifugate was slowly removed. The cells were resuspended and transferred onto a freshly prepared agarose gel surface sample (as described above). The cells were allowed to settle on the surface of the agarose gel for 30 min (before its application to agarose substrates, the bacterial suspension had a concentration of around 0.5, measured at OD600). The drop was then carefully removed, and FDS was executed as described below in the LB medium environment.

The phototrophic sulfur bacteria *C. okenii* were obtained from Lake Cadagno in the Piora Valley (46°33′ N, 8°43′ E) in the southern Swiss Alps following the protocol described in Ref. [59]. They play a key role in the removal of sulfide from shallow sediments and stratified waters [60]. Upon transferring the cells to the laboratory, they were grown and propagated using a Pfennig’s Medium I protocol with periodic spiking of hydrogen sulfide [61]. Cultivation was run under a light/dark photoperiod (16/8 h) with a light intensity of 38.9 μmol $m^{-2}$ $s^{-1}$. After soft shaking, 100 μL of the cell solution was transferred onto the freshly prepared surface of LMP agarose (as described above). The cells were allowed to settle down for 30 min. Subsequently, the drop was carefully removed, and the measurement was executed as described below in the aqueous environment.

### 2.7. Quantification of *C. okenii*’s Adhesion Properties over Its Growth Stages

The evolution of the cell-to-surface adhesion over time was tracked using FDS, conducted after 2 weeks, 10 weeks, and 14 weeks of inoculation of *C. okenii* into Pfennig’s Medium I. Although it is motile in its natural lake ecosystem, under laboratory conditions, *C. okenii* gradually shifts into a sessile lifeform [62]. The physiology-dependent measurements were conducted using an agarose concentration of 2.2%, following the gel preparation protocol described previously. The cell solution was kept in a glass container enclosed with a septum to protect it from contact with air. For each measurement, 0.8 mL of the cell solution was extracted using a 1 mL syringe and needle. The cells were collected either from the surface of the cell solution (top cells) or from the bottom of the glass container (bottom cells) and transferred into a 2 mL Eppendorf tube. After their removal, the cells were centrifuged at 6000 RPM for 180 s. The pellet was carefully aspirated using a 10 μL pipette and finally transferred onto the prepared LMP agarose gel substrate. The sample was then left to stand for 30 min so that the cells could settle on the substrate. The cantilever was wetted with a drop of DI water before installing the cantilever holder. This gentle method minimized the fluid flow which otherwise may have interfered with the measurements within the sample solution. Each measurement series consisted of six measurements. First, three measurements were performed with the top cells. Then, three measurements with the bottom cells were taken. Each series of measurements was completed within 3 days.

### 2.8. Statistical Tests

We conducted pairwise t-tests to compare the differences in adhesion across different agarose concentrations against that for the control sample (agarose conc. of 1.5%). For the statistical analysis, we employed two-sample *t*-tests with the significance level set at p=0.01. Statistical analyses were performed using GraphPad Prism software, v 10.0.

## 3. Results

### 3.1. The Topography of the LMP Agarose Gel Surfaces

**Figure 2:**
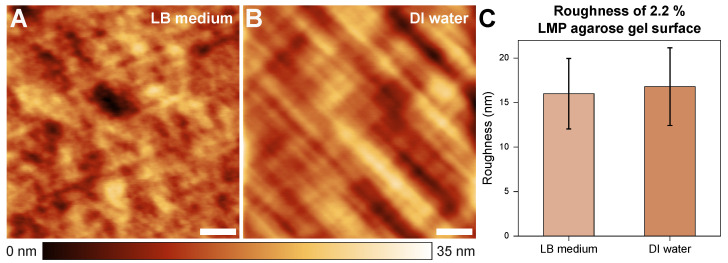
Sample topographies in a 15 × 15 μm² area of samples with a 2.2% LMP agarose gel concentration in LB medium (**A**) and water (**B**) environments. The roughness is 16.01 (±3.96) nm and 16.80 (±4.37) nm, respectively (**C**). The scale bars indicate 3 μm.

Figure 2 shows the topography of 2.2% agarose gel in the LB medium (A) and DI water (B) environments. The sample surfaces showed low surface roughness overall; furthermore, no significant differences in roughness were observed between the two cases.

### 3.2. Mechanical Properties of the LMP Agarose Gels

**Figure 3:**
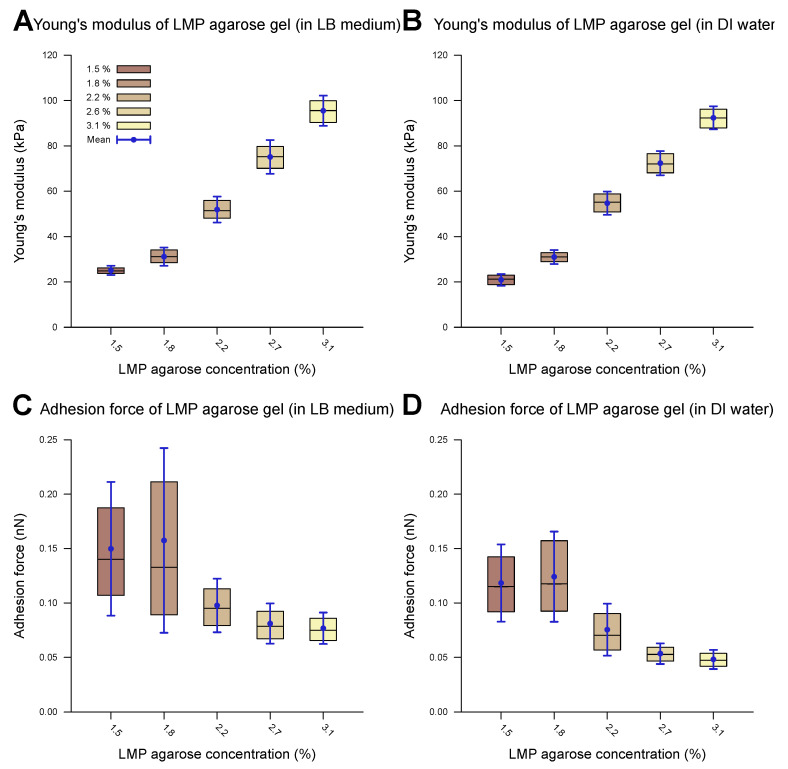
Measured mechanical properties of agarose gel of different concentrations. The inner 50% of the measured values sorted in ascending order is represented by a colored area containing the median as a horizontal line. In addition, the average is shown as a point and the standard deviation as an error bar. The difference between water and the LB medium as the measurement environment is imperceptible. The Young’s modulus of the agarose gel increases with a rising LMP agarose concentration and lies between 20 and 100 kPa in both the LB medium (**A**) and water (**B**) environments. The statistical test (two-sample *t*-test) revealed a significant difference in the Young’s modulus when the agarose concentration was changed from 1.5% to 3.1% for both the LB medium and water (*p*-value < 0.001). Only small adhesion forces within the sub-nN scale could be observed for both the LB medium (**C**) and water (**D**) environments during contact with a spherical SiO_2_ tip (3.5 μm). The adhesion forces decreased slightly with an increasing LMP agarose concentration. The statistical test (two-sample*t*-test) revealed a significant difference when the agarose concentration was changed from 1.5% to 3.1% for both the LB medium and water (*p*-value < 0.01).

A representative example of a measured force–distance curve is given in Figure 1A. The computed outcomes of the Young’s modulus and adhesion values of the agarose gel samples without cells are presented in Table 2. Figure 3 shows the corresponding measurements carried out in the DI water and the LB medium. Overall, the measurements in the DI water and the LB medium show comparable values across the different parameters studied in this work. The provided data include both the median and mean values (n>500).

The Young’s modulus, also known as the modulus of elasticity, is a measure of the stiffness of a material, defined as the ratio of stress to strain within the elastic limit of the material. Upon increasing the LMP agarose concentration, the Young’s modulus increases, for both DI water and LB medium environments. With Young’s moduli smaller than 100 kPa, the agarose substrate falls within the range of soft biomaterials (e.g., cells, organs) and soft synthetic polymers [63,64]. The observed trend can be elucidated by examining the underlying structure of agarose gels [29,65,66]. LMP agarose monomers consist of β-D-galactose and 3,6-anhydro-α-L-galactose units. Upon cooling the agarose gel solution below the gelation temperature (24–28 °C), the equatorial hydrogen atoms of the 3,6-anhydro-α-L-galactose residues form hydrogen bonds with each other, inducing the formation of α-helices, forming a single chain or double chains. The cooling rate determines the ratio of one-chain and two-chain α-helices. This process results in the formation of a 3D secondary agarose gel structure. The gelation temperature and pore size of the gel depend mainly on the availability of hydrogen and oxygen in the agarose solution. A lower agarose concentration leads to fewer α-helices, resulting in reduced structural stability and larger pore sizes. Alongside α-helices, other types of physical cross-linking between the chains increase the stability of the gel matrix. The structural stability directly correlates with the Young’s modulus of the gel, explaining the increase in the Young’s modulus with a higher concentration of agarose. Low-melting-point agarose is produced through hydroxyethylation such that a portion of the available alcoholic side groups is substituted by ethyl groups and hence it is unable to form hydrogen bonds. This substitution reduces the density and strength of the α-helices, leading to a lower Young’s modulus [67]. Overall, the Young’s moduli we measured were lower than those of normal-melting-point agarose gels as reported in the literature [47]. Roberts et al. investigated the elastic storage modulus of 1% normal agarose gel to be around 80 kPa [46], with other reports corroborating their results [47,68,69,70,71].

The mechanical properties of LMP agarose gels in LB medium have received limited investigation so far. Existing studies report varying results on the mechanical properties across different agarose concentrations, though the overall range matches well with our measurements. Kontomaris et al. used a similar LMP agarose gel and measured an elastic modulus of 154 kPa (2.5%) [72], while Topuz et al. observed values in the same range as those in this study for LMP agarose gel [73]. Finally, Zamora-Mora et al. measured a Young’s modulus of 400 Pa for 0.5% agarose gel and 1.45 kPa for a 2% agarose concentration [74], similar to Kumachev et al., who reported the range to be between 50 Pa (0.75%) and 2.5 kPa (3.00%) [75]. It should be noted that the last two studies measured the shear modulus, though the Young’s modulus is expected to be in the same range as well.

### 3.3. The Cell–Surface Adhesion Force on the LMP Agarose Gels

**Figure 4:**
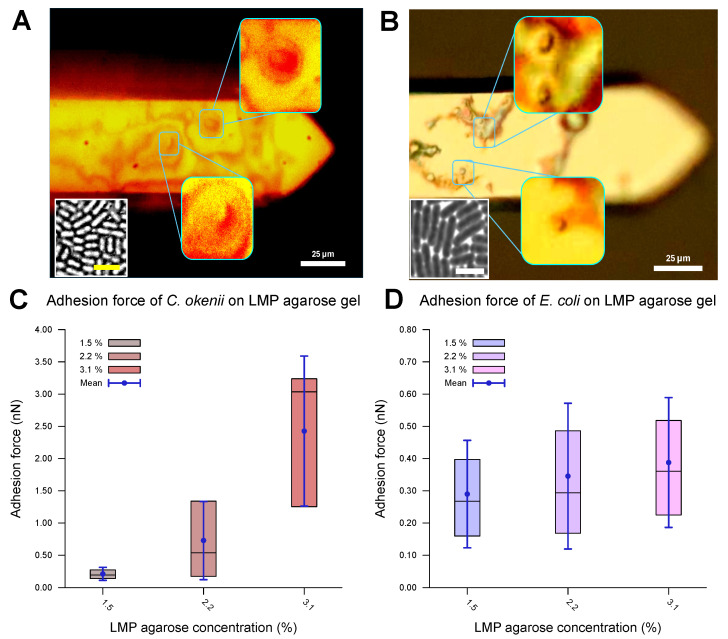
Adhesion properties of *C. okenii* and *E. coli* on agarose gels of varying concentrations. The optical microscope, built onto the atomic force microscope stage, allowed for the visualization of the bacterial cells on the cantilevers, as shown in (**A**) for *C. okenii* and (**B**) for *E. coli*. The presence and viability of the cells on the cantilever were further confirmed by bringing the cantilevers in contact with agarose pads: cells from the cantilever then grew on the agarose plates (insets, with a scale bar of 10 μm), confirming their presence on the cantilever tips. In (**C**,**D**), the inner 50% of the measured values for the force of adhesion to the LMP agarose gel, sorted in ascending order, is represented by a colored area containing the median as a horizontal line. In addition, the average is shown as a point and the standard deviation as an error bar. Interestingly, as the LMP agarose concentration was increased, a substantial increase for the *C. okenii* bacterial cells (**C**) and a slight rise in the adhesion force for the *E. coli* bacterial cells (**D**) were observed. The statistical test (two-sample *t*-test) revealed a significant change in *C. okenii*’s adhesion to the 1.5% versus the 3.1% agarose concentration (*p*-value < 0.01), while for *E. coli*, this difference was not significant.

The evaluated values for the adhesion of the *E. coli* and *C. okenii* cells to the LMP agarose gels with different agarose concentrations are listed in Table 3 and plotted in Figure 4. The optical microscopy images (Figure 4A,B) reveal that the cells were attached to the surface of the cantilever, confirming that the adhesion force between the cell and the agarose surface was measured. The force–distance curves show a smooth adhesion characteristic, as has been observed for the initial adhesion of cells to hydrophilic surfaces [54]. For *E. coli*, the adhesion values are in the weak sub-nN range. As can be seen, adhesion forces slightly increase as concentration of agarose increases, which is in agreement with the adhesion to normal agarose gels [21].

**Figure 5:**
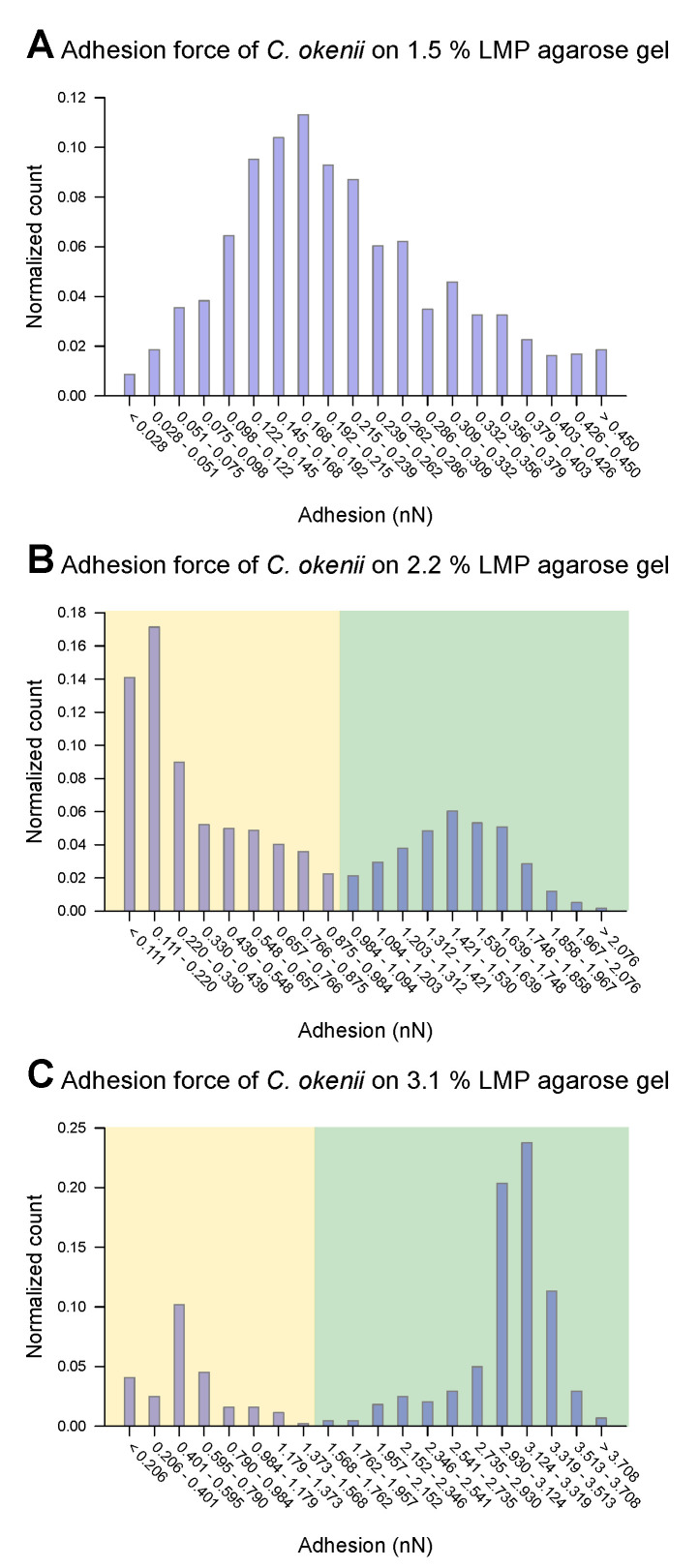
Distribution of force of *C. okenii*’s adhesion to 1.5% LMP agarose gel (**A**), 2.2% LMP agarose gel (**B**), and 3.1% LMP agarose gel (**C**). While for the 1.5% LMP agarose gel the adhesion force is unimodal, two peaks occur for the 2.2% (**B**) and 3.1% (**C**) agarose concentrations. A manual threshold was set to divide the occurring peaks into a lower-adhesion-force part (highlighted in yellow) and a higher-adhesion part (highlighted in green).

For *C. okenii*, however, the adhesion force increased by up to an order of magnitude compared to the adhesion forces for *E. coli*. Furthermore, *C. okenii* was found to be more sensitive to changes in the concentration of LMP agarose. While doubling the agarose concentration from 1.5% to 3.1% increases the median adhesion force on the surface by a factor of 1.3 for the *E. coli* cells, it is enhanced by a factor of 15 for *C. okenii*. Furthermore, a detailed examination of the distribution of the adhesion force data for *C. okenii* reveals the emergence of a bimodal distribution of the adhesion force at an LMP agarose concentration of 2.2%, which then becomes more pronounced when the agarose concentration is raised to 3.1% (see Figure 5). In order to understand the co-existence of the adherent phenotypes, we separated the two peaks in the bimodal adhesion force distribution for the 2.2% and 3.1% agarose concentrations, with dynamic threshold adhesion values, as indicated by the yellow and green regions in Figure 5. The bimodal distributions indicate the co-existence of two different adherent sub-populations—weakly adherent cells (the yellow regions of the plot) and strongly adherent cells (the green regions of the plot)—which emerge as the stiffness of the underlying substrate is modulated. At an LMP concentration of 1.5%, such a bimodality could not be detected.

**Figure 6:**
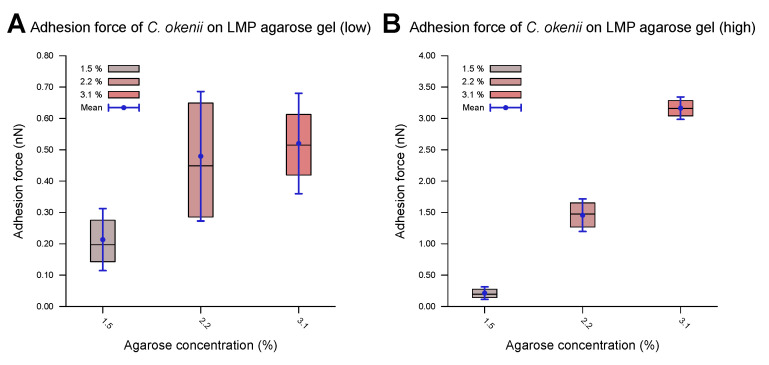
Force of *C. okenii*’s adhesion to LMP agarose gels of various concentrations revealed distinct adhesion regimes. In the low-adhesion-force regime, the adhesion force showed a weak dependence on the agarose concentration (**A**). However, in the higher-adhesion-force regime, a clear dependence on the adhesion force could be observed (**B**); here, the adhesion force increased by an order of magnitude as the agarose concentration varied from 1.5% to 3.1%.

As shown in the boxplots in Figure 6, the lower range of the peaks in the distribution of the force of *C. okenii*’s adhesion is around the same range as that for the *E. coli* cells, i.e., a low adhesion force that depends weakly on the agarose concentration. In contrast, the higher adhesion force peaks seen for *C. okenii* reveal a stronger dependence on the LMP agarose concentration. The low adhesion noted for both *E. coli* and *C. okenii* corresponds with the general observation that bacteria with hydrophobic surfaces tend to adhere more readily to hydrophobic than hydrophilic surfaces. Nevertheless, the weak adhesion forces observed here vary slightly as the concentration of agarose increases. To understand this, a bacterium can be modeled as a solid body, with the biopolymers on its surface represented by flexible chains [54]. During the separation of the cell from the surface, various forces, including electrostatic forces, hydrogen bonds, dipole interactions, and Lifshitz–Van der Waals forces, act to oppose detachment. Taken together, these interactions can be captured using extended DLVO theory (xDLVO) [76]; however, in a more complex biological context (such as the one studied here), the xDLVO theory might require further modifications, which are beyond the scope of this work. Another possible effect during the detachment of a bacterial cell is steric hindrance due to the partial entanglement of the polymeric surface chains [54]. The minimum force required for complete separation corresponds to the adhesion force, which depends on the properties of the flexible surface chains and the structure and properties of the underlying network, which, in turn, alter with the agarose concentration, offering a possible reason for the slight enhancement in the weak adhesion forces with the increase in the agarose concentration.

While *E. coli* showed weak adhesion forces across the different LMP agarose concentrations tested, *C. okenii* showed a different trend. The bimodal distribution indicates that some cells bind strongly to the surface macromolecules, while others bind weakly. Similar behavior has also been observed for *Staphylococcus aureus* on hydrophobic surfaces [54]. Increasing the LMP agarose concentration can provide more anchor points for the *C. okenii* cells, thus increasing the overall adhesion force. However, the exact binding mechanism is currently under investigation and will be discussed elsewhere.

### 3.4. Adhesion of the Polystyrene Microparticles to the LMP Agarose Gels

**Figure 7:**
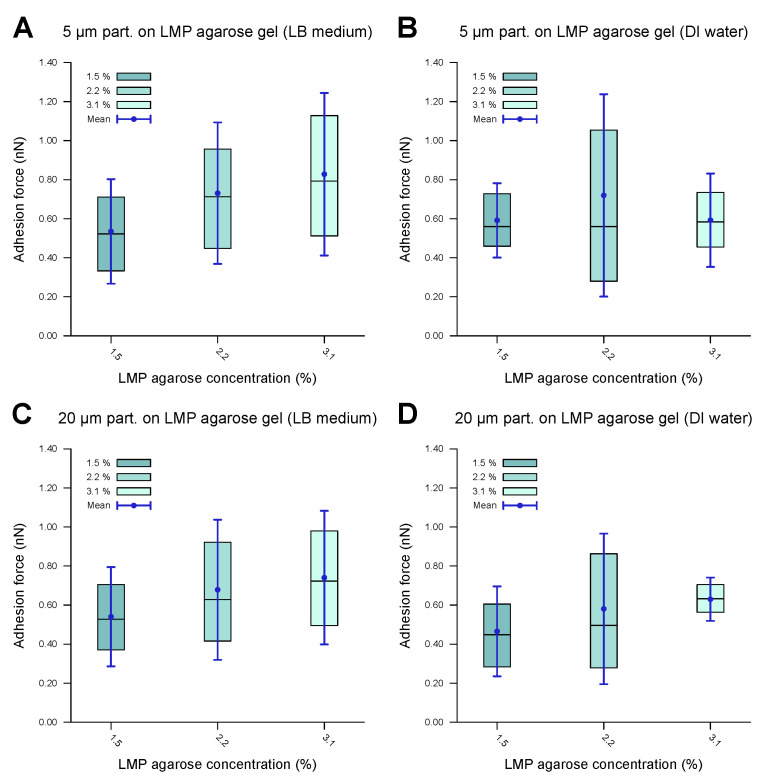
Forces of adhesion of polystyrene microparticles to LMP agarose gels of various concentrations measured in a DI water environment. The inner 50% of the measured values sorted in ascending order is represented by a colored area containing the median as a horizontal line. In addition, the average is shown as a point and the standard deviation as an error bar. (**A**) demonstrates the 5 μm particles showing low to medium adhesion to the LB-medium-based LMP agarose gel, with a slight increase in the adhesion force as the agarose concentration is increased. (**B**) demonstrates the 5 μm particles showing similar behavior for DI-water-based LMP agarose gel to that in (**A**). (**C**,**D**) demonstrates the 20 μm microparticles showing similar behavior for the LB-medium-based LMP agarose gel (**C**) to that of the 5 μm microparticles (**A**), with a similar adhesion force range, and similar behavior for DI-water-based LMP agarose gel (**D**) to that of the 5 μm microparticles (**B**), with a similar adhesion force range. The data indicate comparable values, with no significant difference.

To understand the trends in the adhesion force better and to see whether there was active biological modulation of the adhesion, the experiments were repeated with passive PS microparticles. As with the bacterial cells, the PS microparticles possessed hydrophobic surfaces. To maintain a comparable size range, microparticles with diameters of 5 μm and 20 μm were used. The system also consisted of an agarose gel based on LB medium as the substrate and DI water as the surrounding medium. To minimize the influence of the ions present in the LB medium, these experiments were repeated in a system with agarose gel based on DI water in a DI water environment. The results are shown in Figure 7, while the corresponding values are provided in Table 4. The adhesion forces fall within the nN range, with a marginal increase with the agarose concentration. A comparison with the results for *E. coli* and *C. okenii* shows that the adhesion force for PS beads is somewhat higher. Though our results cannot conclusively confirm whether *E. coli* actively tunes its adhesion to LMP agarose, *C. okenii* exhibits a distinct adhesion trend, suggesting a specific interaction between the substrate and the bacteria that manifests at a specific agarose concentration. This phenomenon aligns with previous findings on the adhesion of *Staphylococcus aureus* [54], suggesting the presence of various active adhesion mechanisms in bacteria.

### 3.5. Evolution of *C. okenii*’s Adhesion over Its Growth Stages

**Figure 8:**
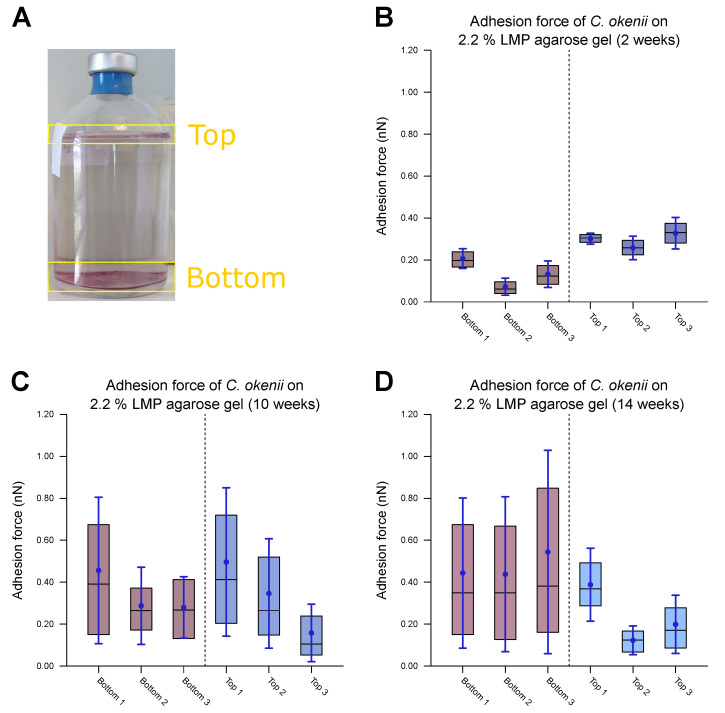
Measurements of force of *C. okenii*’s adhesion to 2.2% LMP agarose gel. (**A**) The freshly harvested *C. okenii* cells were stored in a glass jar under anaerobic conditions exposed to room temperature and daylight. Bottom and top layers of *C. okenii* formed within the vessel. For each chosen time point, three samples of the top layer and three samples of the bottom layer were measured. For (**B**–**D**), the inner 50% of the measured values sorted in ascending order is represented by a colored area containing the median as a horizontal line. In addition, the average is shown as a point and the standard deviation as an error bar. (**B**) Results on the adhesion force of the bottom- and top-layer cells 2 weeks after their removal from their natural habitat, showing low adhesion only. (**C**) Results on the adhesion force of the bottom- and top-layer cells 10 weeks after their removal from their natural habitat, showing an increased adhesion force range for all samples. (**D**) Results on the adhesion force of the bottom- and top-layer cells 14 weeks after their removal from their natural habitat, showing a large adhesion force range, especially for the bottom cells.

As the final step of our experiments, we measured the evolution of *C. okenii*’s adhesion forces during the course of laboratory inoculation. A recent report by the authors has demonstrated that under laboratory conditions, *C. okenii* undergoes a lifeform shift from a free-living, motile to a biofilm-forming, sessile phenotype [62]. Since bacterial adhesion to surfaces is central to biofilm formation, the final measurements were motivated by the following question: Does the lifeform shift in *C. okenii* correspond to changes in its cell–substrate adhesion properties? Regardless of the exact mechanism of adhesion between *C. okenii* and the LMP agarose substrate, it is intriguing that *C. okenii* populations elicit weakly and strongly adherent phenotypes at a higher substrate stiffness. Relative to the planktonic states, with suppressed cell–surface adhesion properties, the transition to sessile lifeforms may be triggered by amplified adhesion mechanisms (for instance, via the secretion of exopolymeric substances under environmental stressors), driving cellular flocculation and surface attachment, ultimately leading to sessile lifeforms [77]. Thus, starting with a fresh sample of planktonic *C. okenii* isolated from its natural lake habitat, we analyzed its cell–substrate adhesion forces and compared them with those in lab-grown cultures over a period of 14 weeks (in the late stationary phase, [62]). Macroscopically, we observed two distinct *C. okenii* layers in the inoculation bottle: a bottom layer attached to the floor of the glass bottom and a top suspended layer at the air–water interface, as visualized and labeled in Figure 8.

**Figure 9:**
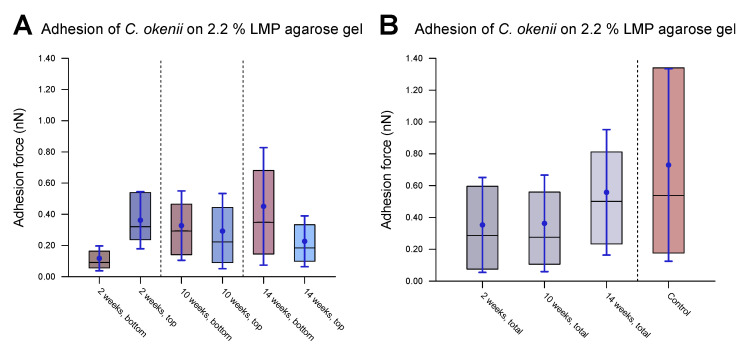
Evolution of *C. okenii* cells’ adhesion to 2.2% LMP agarose gel over time in laboratory conditions. The inner 50% of the measured values sorted in ascending order is represented by a colored area containing the median as a horizontal line. In addition, the average is shown as a point and the standard deviation as an error bar. The plots show the evaluated adhesion force data 2, 10, and 14 weeks after the transfer from the lake to the laboratory. (**A**) shows separated data for the bottom and top cells. As can be seen, the top cells initially show a slightly higher adhesion than that of the bottom cells. (**B**) shows the combined bottom/top cell data as a function of time. For comparison, control cells of the 8th generation in the laboratory are shown on the right. The increase in adhesion with domestication time indicates the growing ability of the cells to form biofilms. Furthermore, the growing adhesion range is an indication of a different development stage either towards or within biofilm formation.

Figure 8B–D illustrate the adhesion values of both the top and bottom cells to a 2.2% agarose gel substrate over time after their removal from their natural habitat and their maintenance under laboratory conditions. A combination of the top and bottom cells from each time period is shown in Figure 9, including data for the populations grown into the late stationary phase (Figure 9B). Table 5 lists the corresponding values.

After two weeks of laboratory inoculation, i.e., when the *C. okenii* population was in its mid-exponential phase, the cell adhesion forces fell within a narrow range, with the *top* cells exhibiting higher adhesion (0.36±0.18 nN) relative to that of their *bottom* counterparts (0.12±0.07 nN). The scenario changes after ten weeks, when the *C. okenii* population enters the stationary phase: the bottom sub-populations have enhanced adhesion, with an overall increase in the diversity of the adherent phenotypes (as indicated by the high standard deviations in our measurements; see Table 5). The difference between the median values of the upper and lower cells decreased. Finally, once the population reached the late stationary phase (at 14 weeks), the bottom population became considerably more adherent, with larger diversity in the adherent phenotypes. The top population, however, maintained its adhesion. A closer examination of the separate measurements for the bottom and top cells revealed a progressive evolution in the adherent characteristics of the bottom cells, whereas the adhesion properties of the top cells appeared to stabilize over the same period of time. Thus, not only do the mean and median adhesion forces increase but the entire range of adhesion forces, including the maximum adhesion force, is also enhanced over time, accompanied by an overall decrease in the number of the *top* cells.

## 4. Discussions and Conclusions

In this work, we present a force–distance spectroscopic approach to simultaneously measuring the stiffness of and adhesion to low-melting-point agarose gel, both in its native state and in the context of the cell–surface adhesion of bacterial species. Our results show that with an increasing agarose concentration, the substrate’s stiffness goes up, remaining below the typical values for normal agarose gels, however. The Young’s moduli of the LMP agarose gels increase from approximately 20 kPa to around 100 kPa as the LMP agarose concentration doubles from 1.5% to 3%. The reported range of Young’s moduli falls between the values for ultralow-melting-point agarose and normal-melting-point agarose, and it thus offers a well-suited alternative to the existing hydrogels used in biomedical research and engineering. No significant differences were observed depending on the choice of liquid environments (LB versus DI water) within which the measurements were conducted. The stiffness of LMP agarose gel lies in the range of soft tissues such as muscle, epithelial, and neural tissues [78], making it a potential material for organ-on-a-chip tissue engineering applications.

The FDS experiments with *E. coli* and *C. okenii* bacteria on LMP agarose gel of three different concentrations revealed a stiffness-dependent variation in the cell-to-surface adhesion. While *E. coli* showed a marginal increase in adhesion with an increasing agarose content, the attachment of *C. okenii* showed two different adherent phenotypes: one with the adhesion force within the same range as that observed for *E. coli* (weak adhesion) and a second sub-population of strongly adherent cells, as well as increased adhesion with an increased LMP agarose concentration. Previous works with diverse species and substrates have indicated inconsistent adhesion forces, underscoring the possibility of cofounding factors, beyond stiffness, contributing to the dynamics of bacteria–substrate adhesion. For example, Thio et al. determined the forces of *E. coli* bacteria’s adhesion to polystyrene, and different polyamides were within the range of 2.9 nN to 9.7 nN [79], while Wang et al. reported that *Staphylococcus aureus*’s adhesion to polyacrylamide hydrogels decreases by three orders with the increasing stiffness of the substrate [80]. While *E. coli* and *P. aeruginosa* show a similar trend in their adhesion to polydimethylsiloxane substrate (0.1 MPa to 2.6 MPa) [22], *E. coli* and *S. aureus* show an increased adhesion to stiffer poly(ethylene glycol) dimethacrylate hydrogels relative to that to softer ones [27,28]. This was also supported by Francius et al. [81], who observed linearly increasing dependence of the adhesion of different *E. coli* strains to poly(allylamine hydrochloride)/hyaluronic acid hydrogels on stiffnesses between 20 kPa and 700 kPa. Guégan et al. [21] measured the effect of the stiffness of agarose gel of two different concentrations (0.75% and 3.0%) on the adhesion capability of Gram-negative *Pseudoalteromonas* sp. D41 and Gram-positive *Bacillus* sp. 4J6 using retention assays. While *Pseudoalteromonas* sp. D41 showed higher adhesion to a softer agarose substrate, the adhesion of *Bacillus* sp. 4J6 increased with increasing stiffness. Although the role of the substrate’s rigidity for motile bacteria has been discussed, specifically in the context of early biofilm development [82,83], further studies will be needed to explore the potential molecular mechanisms underlying stiffness-dependent adhesion in sessile bacteria and how this attribute emerges across the physiological growth stages of the species.

**Figure 10:**
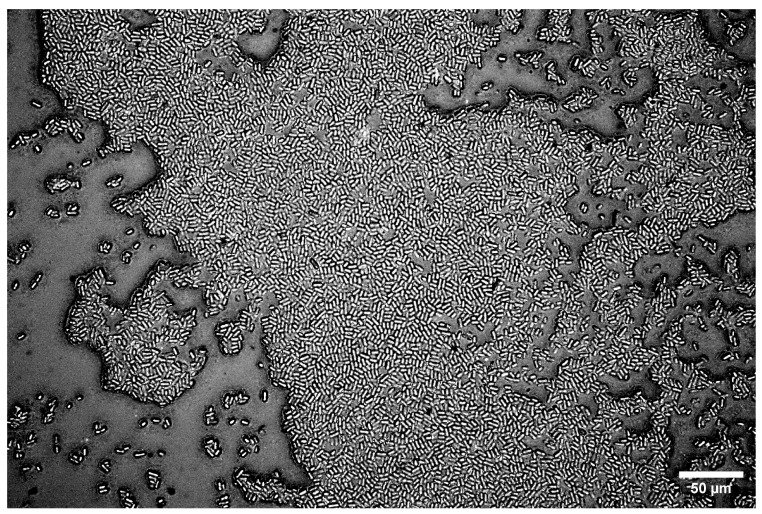
*C. okenii* biofilm on 2.2% LMP agarose gel for a 14-week-old culture. The cells form a monolayer on the underlying LMP agarose substrate.

The mechanism of this adhesion process depends on the properties of the bacteria, the substrate, and the local environment [10,84,85]. For *C. okenii*, a larger contact area of surface polymeric molecules on the substrate, as well as its surface chemistry, may promote the observed adhesion and adherent diversity on agarose gel substrates. Our experiments across *C. okenii*’s physiological growth stages indicate a progressive increase in adhesion (to the 2.2% agarose gel) over time, supporting the lifeform shift from the planktonic to the biofilm state [62]. Biofilm formation by *C. okenii* could be observed stably after 14 weeks of inoculation, as shown in Figure 10. As reported previously by Di Nezio et al. [62], such shifts in lifeform triggered by the domestication of the species occur synergistically across multiple phenotypic traits, including morphology, cell density, intracellular attributes, and alterations in motility. The cell–substrate adhesion data support the previously reported shift in the *C. okenii* lifeform, occurring over a period of weeks after laboratory inoculation. The phenotypic diversity in the adherent cells increases over time, furthermore suggesting an adaptive transformation that could allow the domesticated cells to optimize their surface attachment once they have been inoculated for extended periods under laboratory conditions. Alterations in cell adhesion following domestication have also been reported for *Saccharomyces cerevisiae* [86], wherein such changes were mediated by the modified expression of genes related to cell–cell adhesion and the production of EPS [86,87]. Similar pathways could be targeted in follow-up studies to delineate how planktonic *C. okenii* undergoes lifeform shifts, specifically in the context of stiffness- and physiology-dependent cell adhesion. This study offers biophysical insights into the role of substrate rigidity in bacterial–surface adhesive interactions, with potential applications in both biomedical and environmental fields. Understanding how bacterial growth and adhesion vary with substrate stiffness could inform the design of smart anti-fouling surfaces, optimize biomaterial development, and contribute to our mechanistic understanding of ecological settings in which bacterial dynamics play a critical role. It will be valuable to conduct future investigations, including ones that take the role of hydrophobicity (and hydrophilicity), as well as the strain- and species-specific variability in adhesion, into account. Our results highlight that the stiffness-mediated adhesive interactions depend on the lifestage of a bacterial colony, and thus, the physiological growth phase should be taken into account while analyzing and interpreting bacteria–surface interactions. Additional experimental validation using different bacterial species, though it is beyond the scope of the current study, will be an important step toward assessing the generality of the results presented here.

## Figures and Tables

**Table 1:** Agarose concentrations.

[g/mL]	[%]
0.0150	1.5
0.0183	1.8
0.0221	2.2
0.0262	2.6
0.0306	3.1

**Table 2:** Young’s modulus of agarose gel samples with different agarose concentrations.

	Concentration	Young’s Modulus		Adhesion Force		Number of Measurements (*n*)
	[%]	Average [kPa]	Median [kPa]	Average [nN]	Median [nN]	
LB Medium	1.5	25.1 (±2.1)	24.8	0.15 (±0.06)	0.14	881
	1.8	31.1 (±4.0)	31.1	0.16 (±0.08)	0.13	1083
	2.2	51.9 (±5.7)	51.4	0.10 (±0.02)	0.10	1069
	2.6	75.1 (±7.5)	75.2	0.08 (±0.02)	0.08	589
	3.1	95.5 (±6.7)	95.6	0.08 (±0.01)	0.07	529
Water	1.5	20.9 (±2.6)	21.2	0.12 (±0.03)	0.11	557
	1.8	31.0 (±3.1)	31.0	0.13 (±0.04)	0.12	589
	2.2	54.7 (±5.1)	55.2	0.08 (±0.02)	0.07	931
	2.6	72.4 (±5.3)	72.0	0.05 (±0.01)	0.05	522
	3.1	92.4 (±5.1)	92.3	0.05 (±0.01)	0.05	503

**Table 3:** Adhesion force of bacteria to agarose gels of different concentrations.

Strain	Concentration [%]	Average [nN]	Median [nN]	Measurements
*E. coli*	1.5	0.29 (±0.17)	0.27	482
*E. coli*	2.2	0.35 (±0.23)	0.29	722
*E. coli*	3.1	0.39 (±0.20)	0.36	917
*C. okenii*	1.5	0.21 (±0.10)	0.20	862
*C. okenii*	2.2	0.73 (±0.60)	0.54	1476
*C. okenii*	3.1	2.42 (±1.16)	3.04	443

**Table 4:** Adhesion of polystyrene microparticles to LPM agarose gels.

Agarose Gel Medium	Particle	Agarose	Adhesion Force		Number of Measurements (*n*)
	Diameter [μm]	Concentration [%]	Average [nN]	Median [nN]	
LB medium	5	1.5	0.54 (±0.27)	0.52	902
LB medium	5	2.2	0.73 (±0.37)	0.72	475
LB medium	5	3.1	0.83 (±0.41)	0.80	466
LB medium	20	1.5	0.45 (±0.25)	0.53	474
LB medium	20	2.2	0.68 (±0.36)	0.63	429
LB medium	20	3.1	0.74 (±0.34)	0.72	256
DI water	5	1.5	0.59 (±0.19)	0.56	389
DI water	5	2.2	0.72 (±0.52)	0.56	390
DI water	5	3.1	0.59 (±0.23)	0.59	300
DI water	20	1.5	0.47 (±0.23)	0.45	432
DI water	20	2.2	0.58 (±0.39)	0.50	319
DI water	20	3.1	0.63 (±0.12)	0.64	387

**Table 5:** Adhesion of *C. okenii* to LMP agarose gel across growth stages.

Time Since Inoculation [Weeks]	Extraction Point	Adhesion Force		Number of Measurements (*n*)
		Average [nN]	Median [nN]	
2	Bottom	0.12 (±0.07)	0.09	1479
2	Top	0.36 (±0.18)	0.32	1310
10	Bottom	0.33 (±0.23)	0.29	1940
10	Top	0.23 (±0.24)	0.22	2042
14	Bottom	0.45 (±0.37)	0.35	2338
14	Top	0.23 (±0.16)	0.19	1922

## Data Availability

All of the data and supporting materials are included in the manuscript. Any additional supporting material for this study can be obtained from the corresponding author upon reasonable request.
